# *Leishmania infantum* in red foxes (*Vulpes vulpes*): from clinical findings to cytokine expression

**DOI:** 10.1186/s13071-026-07334-z

**Published:** 2026-03-23

**Authors:** Mario H. Alves, Mariaelisa Carbonara, Natalizia Palazzo, Floriana Gernone, Viviane Noll Louzada-Flores, Antonio Camarda, Michela Prioletti, Filipe Dantas-Torres, Marcos Antonio Bezerra-Santos, Jairo Alfonso Mendoza-Roldan, Domenico Otranto

**Affiliations:** 1 Department of Veterinary Medicine, Università degli Studi di Bari Aldo Moro, Valenzano, Italy; 2 National PhD Program in One Health Approaches to Infectious Diseases and Life Science Research, Department of Public Health, Experimental and Forensic Medicine, University of Pavia, Pavia, Italy; 3 Unité de Parasitologie Moléculaire et Signalisation, INSERM U1201, Institut Pasteur, Université Paris Cité, Paris, France; 4 Aggeu Magalhães Institute, Fundação Oswaldo Cruz (Fiocruz), Pernambuco, Brazil; 5 Department of Veterinary Clinical Sciences, City University of Hong Kong, Hong Kong, China

**Keywords:** Epidemiology, Leishmaniosis, Parasitology, Wildlife, Zoonosis

## Abstract

**Background:**

The life cycle of *Leishmania infantum* is maintained mainly in dogs in anthropogenic environments and in many other wild animals in the sylvatic cycle. The ecological plasticity of some wild canids facilitates their role as hosts for *Leishmania* spp. in different endemic regions. Although red foxes (*Vulpes vulpes*) frequently test positive for *L. infantum* in Europe, little is known about their clinical presentation, immune response, or treatment outcomes. This study investigated the prevalence, clinical, and immunological features of *L. infantum* infection in foxes from southern Italy, complemented by an in vitro evaluation of cytokine responses in fox macrophages.

**Methods:**

Wild foxes from a wildlife rehabilitation center in southern Italy were molecularly and serologically screened for *L. infantum*. One sick fox underwent a complete diagnostic confirmation, treatment, and follow-up through hematological, biochemical, cytological, and molecular evaluations. In addition, peripheral blood mononuclear cells (PBMCs) isolated from a healthy fox were differentiated into macrophages and experimentally infected with *L. infantum* to assess early host–parasite interactions and cytokine gene expression profiles at two time points (4 h and 48 h).

**Results:**

Overall, 13 out of 54 foxes (24.1%) were molecularly positive for *L. infantum*, with a prevalence of 25% (5/20) in necropsied animals and 22% (9/41) in live animals. One individual tested seropositive for *L. infantum* and *Ehrlichia* sp. using the SNAP Leish 4Dx^®^ test (2.4%, 1/41). The sick fox treated with meglumine antimoniate and allopurinol showed marked clinical and laboratory improvement. In the in vitro evaluation, the percentage of infected macrophages decreased from 32.8% at 4 h (2.25 parasites/cell) to 21.5% at 48 h (2 parasites/cell). Cytokine gene expression at 4 h and 48 h showed an increase in interleukin-6 (IL-6) (0.164–0.552) and IL-10 (0.828–4.245), stable IL-4 (0.505–0.708), a decrease in IL-12 (1.793–1.223) and IFN-γ (1.507–0.613), and consistently low TNF-α (0.377–0.411).

**Conclusions:**

The present study confirmed a high *L. infantum* prevalence of infection in red foxes from southern Italy. Serological findings herein and in the literature, together with in vitro cytokine gene expression, suggested that most foxes may remain subclinically infected. The high molecular positivity and the synanthropic nature of red foxes reinforce their role as reservoirs of *L. infantum* in endemic areas.

**Graphical Abstract:**

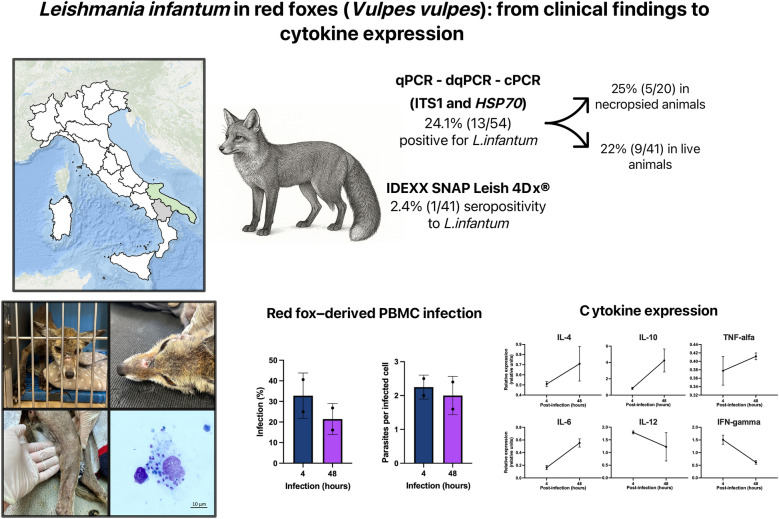

**Supplementary Information:**

The online version contains supplementary material available at 10.1186/s13071-026-07334-z.

## Background

The life cycle of *Leishmania infantum* is perpetuated by phlebotomine sand flies, which transmit the parasite by feeding on different animals, mainly domestic dogs in anthropogenic environments [1]. However, in sylvatic environments, the transmission cycle of this protozoan is maintained by other animal species (e.g., wild canids, lagomorphs, and rodents) [2]. From the Mediterranean basin and African savanna ecosystems to the Asian steppes and the tropical rainforests of Central and South America, several wild canids act as hosts for *L. infantum*, often living near domestic dogs and humans. This is the case for the red fox (*Vulpes vulpes*), the most widely distributed wild carnivoran worldwide [3], followed by the golden jackal (*Canis aureus*) in Africa and Asia [4], the crab-eating fox (*Cerdocyon thous*) in South America [5], and the coyote (*Canis latrans*) in Central and North America [6]. The ecological plasticity of these wild canids facilitates their role as hosts for *Leishmania* spp. in endemic regions [7].

In a pioneering investigation conducted more than 50 years ago in France, red foxes were found to be naturally infected with *L. infantum* on the basis of cytological examination of multiple organs (i.e., blood, liver, spleen, lung, and bone marrow) [8]; this finding was further confirmed in experimental infections of live animals [9]. Overall, investigations of *L. infantum* in red foxes were largely based on necropsied animals [10, 11], with molecular positivity values ranging from 1.3% to 74.6% in Portugal [12] and Spain [13], respectively. Despite reports of *L. infantum* in red foxes [10, 11], no information is available on clinical presentation, immunological findings, or treatment protocols.

Understanding the immune response to *L. infantum* in susceptible animal host species is essential for defining prognosis and disease outcomes [14]. Indeed, cytokine expression patterns may vary considerably among hosts [15]. For example, a dominant Th1-type cytokine response, characterized by the production of interferon-gamma (IFN-γ), tumor necrosis factor-alpha (TNF-α), and interleukin (IL-12), is associated with protection against *L. infantum* infection in dogs because these cytokines activate macrophages toward an M1 phenotype, enhancing intracellular parasite killing [16]. Conversely, disease progression is associated with a shift toward a Th2 response, characterized by the production of regulatory cytokines (e.g., IL-4, IL-6, and IL-10), which promote antibody production, suppress macrophage activation, and favor parasite survival [17]. In addition, circulating immune complexes contribute to tissue inflammation [18]. Although clinical leishmaniosis has been reported in wild carnivorans, such as the crab-eating fox (*Cerdocyon thous*) [19], the Eurasian otter (*Lutra lutra*) [20], wolf (*Canis lupus*) [21], and European mink (*Mustela lutreola*) [22], data on the treatment of leishmaniosis and cytokine gene expression patterns in wild animals remain largely unexplored.

In this study, we investigated the prevalence of *L. infantum* infection in a population of wild foxes in an endemic area of southern Italy. We also reported a case of *L. infantum* infection in a sick fox, described a successful treatment protocol, and assessed experimental infection of red fox peripheral blood mononuclear cells (PBMCs) with *L. infantum* and the associated key cytokine gene expression profiles.

## Methods

### Animal sampling and *Leishmania* detection

**Fig. 1:**
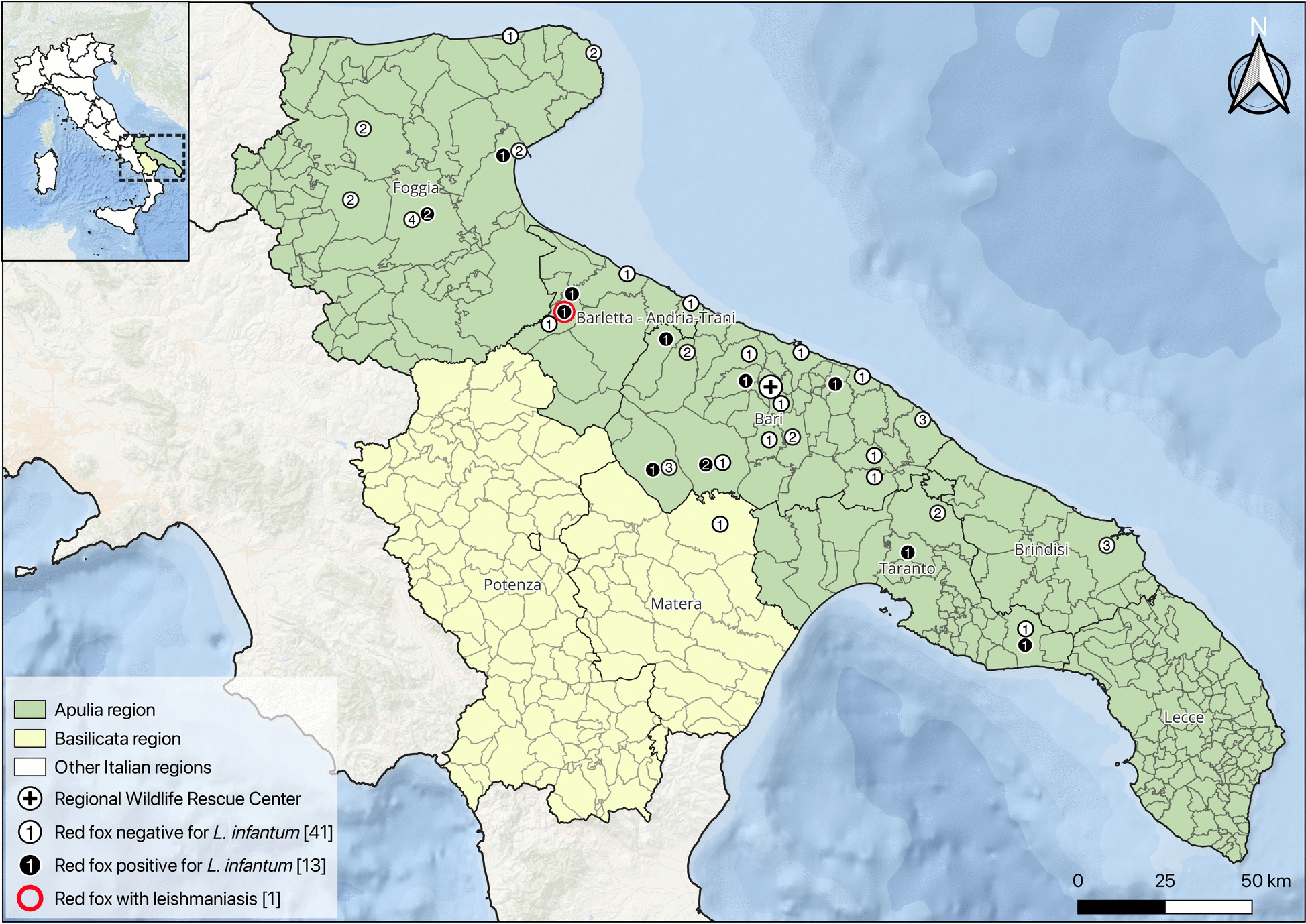
Map of Italy showing Apulia and Basilicata regions where red fox (*Vulpes vulpes*) samples were collected. Each circle represents an individual sampled animal, with white circles indicating negative and black circles indicating positive results for *Leishmania infantum*. When points overlap, the number displayed indicates the total number of samples. The map was prepared in QGIS (version 3.42 Münster) using ESRI^®^ Ocean Basemap imagery

Between August 2024 and October 2025, live red foxes (*n* = 41) from the Basilicata and Apulia regions (southern Italy) were admitted to the Wildlife Rescue Center in Bitetto (Apulia). For each animal, age (i.e., juveniles < 1 year and adults > 1 year) [23], sex, and health status were recorded in individual clinical files as part of the routine diagnostic assessment. Approximately 2 mL of blood was collected from the cephalic or lateral saphenous vein using only physical restraint and transferred into EDTA and serum tubes. In addition, 20 foxes were necropsied (i.e., 13 animals were already dead upon arrival, and 7 died during the rehabilitation). Carcasses were stored at 4 °C for no longer than 72 h or frozen at −20 °C and thawed prior to examination. A complete postmortem evaluation was performed on each fox, and spleen and lymph node samples were used for molecular analyses. In total, 54 red foxes, comprising both live and necropsied individuals (Fig. 1) (Additional file 1), were sampled as part of a previous study [24].

DNA was extracted from blood samples, spleen, and lymph node tissues using commercial kits (DNeasy Blood & Tissue Kit, Qiagen, Hilden, Germany), following the manufacturer’s instructions for each sample type. Subsequently, DNA samples were tested by quantitative polymerase chain reaction (qPCR) for the detection of a *Leishmania donovani* complex kDNA minicircle fragment [25], as well as a duplex quantitative polymerase chain reaction (dqPCR) for the detection and differentiation of *L. infantum* and *Leishmania tarentolae* [26]. Samples with positive qPCR/dqPCR results were subsequently analyzed in duplicate to ensure result consistency, and blank extractions were included in all batches to monitor for potential contamination. Following this, conventional polymerase chain reaction (cPCR) assays targeting *Leishmania* ITS1 [27] and *HSP70* [28] genes were performed on samples that tested positive by qPCR, dqPCR, or both. The cPCR products were analyzed on 2% agarose gels stained with GelRed (VWR International PBI, Milan, Italy) and visualized using a ChemiDoc Touch Gel Imaging System (BioRad, CA, USA). Negative controls (ultrapure sterile water) and positive controls were included in all PCR runs. Amplicons were purified and sequenced in both directions using the same primers as for PCR amplification, with the BigDye Terminator v3.1 chemistry on a 3130 Genetic Analyzer (Applied Biosystems, Foster City, CA, USA). Resulting sequences were edited and analyzed using BioEdit software (version 7.7) [29] and compared with those available in the GenBank database through the Basic Local Alignment Search Tool (BLAST; http://blast.ncbi.nlm.nih.gov/Blast.cgi) for species identification.

The sequences were individually aligned with closely related species retrieved from GenBank using the MUSCLE algorithm in MEGA12 [30]. The evolutionary history of *Leishmania* spp. was reconstructed using the maximum likelihood method. The Tamura 3-parameter model [31] and the Hasegawa–Kishino–Yano model [32] were used for the phylogenetic analyses of ITS1 and *HSP70*, respectively. Both phylogenetic trees included a proportion of invariant sites (+I) and were supported by 2000 bootstrap replicates.

In addition to the molecular diagnosis, all samples from live foxes were screened for *L. infantum*, *Ehrlichia* sp., *Anaplasma phagocytophilum/platys*, and *Dirofilaria immitis* using the SNAP Leish 4Dx^®^ test (IDEXX Laboratories, Westbrook, ME, USA), an ELISA-based rapid test performed directly on whole blood samples according to the manufacturer’s instructions. Blood smears were prepared for all individuals and examined for the presence of hemoparasites using Diff-Quik^®^ staining (Merck, Darmstadt, Germany) [33], followed by microscopic evaluation under a light microscope (Axioscope 5 equipped with an Axiocam 305 color camera, Carl Zeiss Microscopy GmbH, Jena, Germany).

### Clinical case

**Fig. 2:**
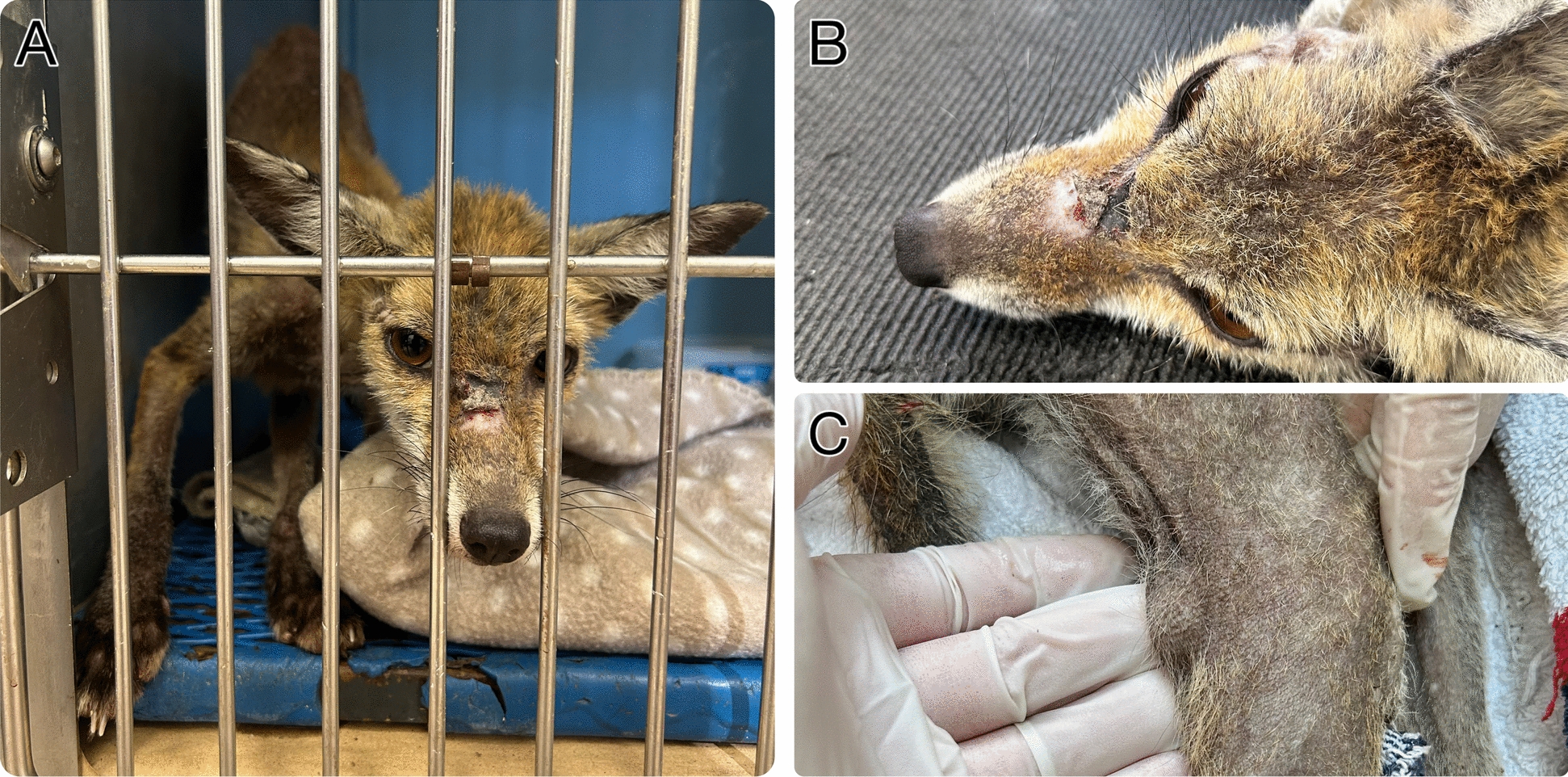
Red fox (*Vulpes vulpes*) with sarcoptic mange and leishmaniosis (**A**). The animal appeared emaciated and cachectic, with ulcerative skin lesions (**B**) and generalized lymphadenopathy (**C**)

During the study, an adult female fox, rescued in Canosa di Puglia (Apulia region), was referred to the Wildlife Rescue Center showing clinical signs suggestive of active leishmaniosis and sarcoptic mange (Fig. 2). After confirming the diagnosis of leishmaniosis through molecular, cytological (i.e., fine-needle aspiration from an enlarged lymph node), and serological tests, a treatment protocol and follow-up plan were established. Hematological, biochemical, and molecular assessments were performed on days 0, 33, and 68 following the animal’s admission, along with serological testing using indirect fluorescent antibody test (IFAT) [34]. Serum protein electrophoresis was carried out using the Helena V8 system (Helena Laboratories, USA), with Dupleix et al. [35] as a reference for values.

Specifically, hematological analyses were performed on the Advia 2120i analyzer (Siemens Healthineers, Germany), while biochemical parameters were measured on the Beckman Coulter AU5800 analyzer (Beckman Coulter, USA). The results were interpreted using reference intervals established for rescued red foxes in Italy [36]. For parameters not reported in that study, additional reference data were retrieved from the Species360 ZIMS database [37]. Manual cell counts were performed at random to verify the accuracy of the automated results. IFAT was performed using promastigotes of *L. infantum* zymodeme MON1 as antigen; serum was diluted 1:80, incubated with the antigen, and then with fluorescein-labeled rabbit anti-dog IgG (1:40; Sigma-Aldrich, Germany) at 37 °C for 30 min, with samples showing cytoplasmic or membrane fluorescence at 1:80 considered positive and further titrated to endpoint [34].

### In vitro experiments

#### Red fox PBMC preparation and infection

An adult male red fox, negative for *Leishmania* spp. by molecular assay (qPCR performed on blood samples), IFAT, and the SNAP Leish 4Dx^®^ test, served as a blood donor for PBMC isolation. Briefly, a total of 6 mL of blood was drawn from the jugular vein into EDTA tubes, and peripheral blood mononuclear cells were obtained and seeded with 100 ng/mL of recombinant human M-CSF (Lyophilized; REF PHC9501, Gibco, Thermo Fisher Scientific) in 24-well plates following previously described procedures [38, 39].

After 5 days of cell maturation and adhesion to the plates, late stationary-phase promastigotes isolated from a dog (MCAN/IT/2025/Max; University of Bari, Italy) at low passage (P3) were washed three times in sterile phosphate-buffered saline (PBS), resuspended in RPMI-1640 medium, and counted. The concentration was adjusted to a multiplicity of infection (MOI) of 10:1 (parasites:cells). A volume of 200 µL of the parasite suspension was added to each well for internalization after medium removal. Plates were incubated (4 h, at 37 °C, 5% CO_2_), and wells were washed three times with PBS to remove noninternalized promastigotes. Intracellular parasites were assessed at 4 and 48 h post-infection in two replicates. Coverslips were stained by Diff-Quik and examined under bright-field microscopy at 40× magnification. Parasite internalization was quantified by counting 100 cells (or the maximum number available when fewer were present) to determine both the percentage of infected cells and the number of parasites per cell, with the final value representing the median of the duplicates for each time point.

#### Cytokine gene expression analysis

Scraped cells from each infection time point were centrifuged, and RNA was extracted using RNeasy Mini Kit^®^ (Qiagen) following the manufacturer’s instructions. cDNA was synthesized using the SuperScript^®^ IV VILO Master Mix (Thermo Fisher Scientific). Cytokine gene expression (IL-6, IL-10, IL-4, IL-12, TNF-α, and IFN-γ) was quantified by qPCR using the SsoAdvanced™ Universal Supermix (BioRad) and PrimePCR^®^ assays, with the same housekeeping gene [40–42] and analytical procedures described in [39]. Relative expression was determined using the 2^−ΔΔCq^ method [43]. Gene expression levels were reported as mRNA relative units, calculated as the ratio of gene expression of infected versus uninfected macrophages, with the final value representing the median of the two duplicates at each time point.

## Results

Of the 54 red foxes examined (*n* = 31 males and *n* = 23 females; *n* = 35 juveniles and *n* = 19 adults), 24.1% (13/54; 95% CI 14.6–36.9) scored positive for *L. infantum* by qPCR targeting kDNA, with most of them being juveniles (76.9%, 10/13; 95% CI 49.7–91.8). The molecular prevalence of infection in necropsied animals was 25.0% (5/20; 95% CI 11.2–46.9) and in live animals 22.0% (9/41; 95% CI 12.0–36.7). None of the blood smears examined showed amastigotes of *Leishmania* spp.

Among the qPCR of *L. infantum*-positive foxes (24.1%), two also tested positive for ITS1 and one for the *HSP70* gene by cPCR. One individual from the above-mentioned animals (i.e., the fox with clinical leishmaniosis) tested seropositive for *L. infantum* and *Ehrlichia* sp. using the SNAP Leish 4Dx^®^ test (2.4%, 1/41; 95% CI 0.1–12.9%) (Table 1). In addition, 4 of 13 positive foxes were co-infected with *Babesia vulpes*, as determined by both molecular diagnostics and blood smear cytology [24].

**Table 1:** Molecular and serological prevalence of *Leishmania infantum* in red foxes (*Vulpes vulpes*) according to age, sex, and sampling category (live animals, individuals that died during rehabilitation, or those found dead upon arrival)

Variables	Total foxes, *n* = 54 (%)	Molecular positivity (%)	Seropositivity
Age			
Juvenile	35 (64.8)	10 (28.6)	0
Adult	19 (35.2)	3 (15.8)	1 (5.2)
Sex			
Female	23 (42.6)	10 (43.5)	1 (4.3)
Male	31 (57.4)	3 (9.7)	0
Sampling category			
Alive animals	34 (63)	8 (23.5)	1 (2.9)
Dead during rehabilitation	7 (13)	1 (14.3)	0
Dead upon arrival	13 (24)	4 (30.8)	–
Total prevalence	13/54 (24.1%, 95% CI 14.6–36.5%)		

In necropsied foxes, the spleen was the most frequently positive tissue for *L. infantum* (*n* = 5), followed by lymph nodes (*n* = 3). One live individual scored positive at whole blood examination, as well as in both spleen and lymph node tissues, following its death and necropsy. The spatial distribution of positive/negative foxes across the study area is shown in Fig. 1.

**Fig. 3:**
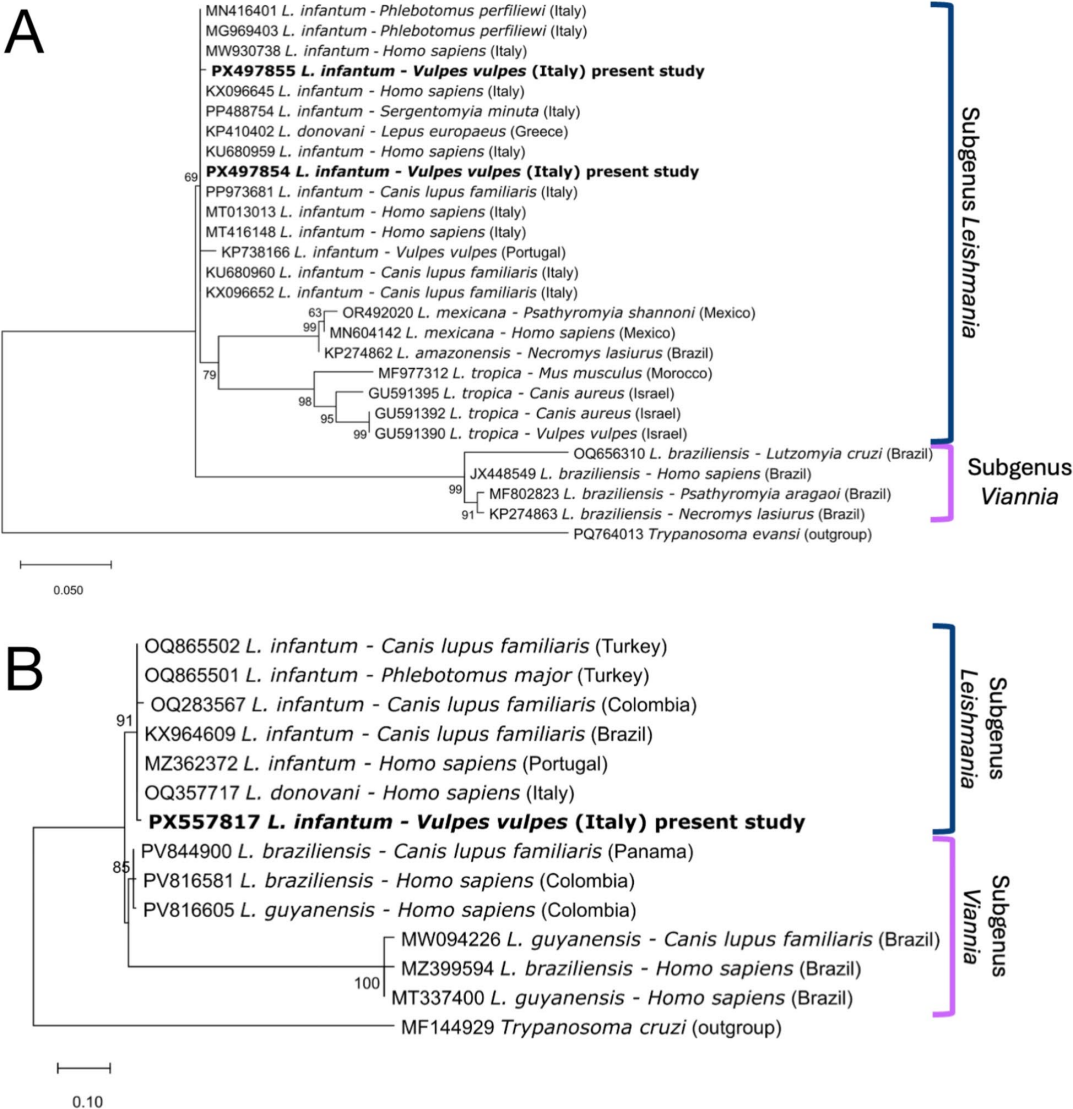
Phylogenetic trees of *Leishmania* spp. based on ITS1 (**A**) and *HSP70* (**B**) gene sequences. Trees were inferred using the maximum likelihood method with 2000 bootstrap replicates. The Tamura 3-parameter model with a proportion of invariant sites (+I) was applied for ITS1, and the Hasegawa–Kishino–Yano model with a proportion of invariant sites (+I) was used for *HSP70*. Sequences from the present study are shown in bold and are labeled with their GenBank accession numbers, host species, and geographic origin

ITS1 and *HSP70* sequences derived from lymph node tissue of a fox with clinical leishmaniosis showed 99.01% and 99.61% nucleotide identity, respectively, with *L. infantum* (GenBank accession numbers MN648748 and MF137824), while the ITS1 sequence obtained from a whole blood sample of the asymptomatic fox showed 99.68% nucleotide identity with *L. infantum* (GenBank accession number OP724554). Accordingly, the phylogenetic analysis of both ITS1 (Fig. 3A) and *HSP70* (Fig. 3B) sequences confirmed a close relationship between the *L. infantum* sequences obtained in this study and those previously reported from humans, dogs, sand flies, and the European hare, as well as the only other sequence available from a red fox from Portugal (Fig. 3A). All these clustered together in a species-specific clade, clearly separated from other species within the same subgenus *Leishmania* and even more distant from those belonging to the subgenus *Viannia*. Representative sequences herein generated were deposited in GenBank (accession numbers PX497854 and PX497855 for ITS1 and PX557817 for *HSP70*).

### Clinical case

One adult female fox showed clinical signs suggestive of sarcoptic mange (i.e., intense pruritus, alopecia, and crusted skin), which was confirmed by a superficial skin scraping. On the day of admission, the fox received a single subcutaneous dose of ivermectin (0.2 mg/kg; Ivomec^®^, Boehringer Ingelheim) and showed dermatological improvement thereafter. In addition, the animal presented apathy, cachexia, pale mucous membranes, disseminated alopecia associated with desquamative and ulcerative skin lesions, and generalized peripheral lymphadenomegaly suggestive of active leishmaniosis (Fig. 2).

**Fig. 4:**
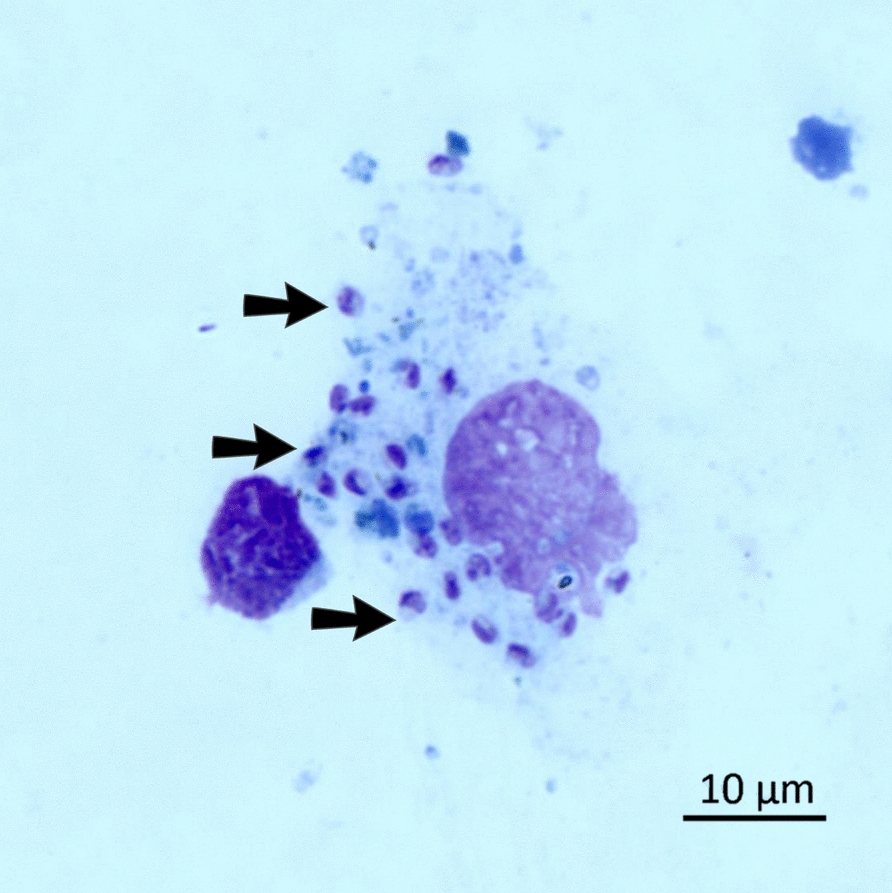
Amastigotes of *Leishmania infantum* (indicated by black arrows) observed in a lymph node smear from a leishmaniotic red fox (*Vulpes vulpes*), stained with Diff-Quik and visualized under a light microscope (Axioscope 5 equipped with an Axiocam 305 color camera, Carl Zeiss Microscopy GmbH, Jena, Germany). Scale bar = 10 µm

On day 0, the complete blood count was suggestive of regenerative anemia [red blood cells (RBC) 5.5 × 10^6^/µL; hemoglobin (HGB) 5.7 g/dL; hematocrit (HCT) 25%; mean corpuscular volume (MCV) 46 fL; mean corpuscular hemoglobin (MCH) 10.5 pg; and mean corpuscular hemoglobin concentration (MCHC) 22.5 g/dL], with a mixed red cell population (i.e., macro- and microcytosis, polychromasia, hypochromia, anisocytosis) and lymphopenia (868/µL). The serum biochemical profile showed normal total proteins (6.8 g/dL), severely decreased albumin (2 g/dL), increased globulins (4.8 g/dL), a low albumin-to-globulin ratio (0.4), hypergammaglobulinemia (26.5%), hyperbetaglobulinemia (28.9%), and elevated C-reactive protein (CRP; 3.4 mg/L) (Table 2). The suggestive clinical and laboratory abnormalities were further confirmed by qPCR on both whole blood and lymph node aspirate (cycle threshold (Ct) values 27 and 17, respectively) as well as by the presence of amastigotes at the cytological evaluation of fine-needle aspiration from the enlarged lymph node (Fig. 4). Serologically, this individual scored positive both by IFAT with a high titer (1:10,240) and by SNAP Leish 4Dx^®^ test, showing reactivity to both *L. infantum* and *Ehrlichia* sp.

**Table 2:** Complete follow-up of the leishmaniotic female red fox (*Vulpes vulpes*), comprising treatments, body weight, hematology, biochemistry, molecular, and serological analyses performed at the initial veterinary examination (day 0) and at the subsequent time points on days 33 and 68

Exams	First exam (4 September 2025/day 0)	Second exam (7 October 2025/day 33)	Final exam (11 November 2025/day 68)	Reference range
Body weight (kg)	2.6	2.8	3.1	3.6–6.5 [77]
Lymph node cytology	Positive for *L. infantum* amastigotes	Negative	Negative	
SNAP Leish 4Dx^®^ (ELISA)	Positive for *L. infantum* and *Ehrlichia* sp.	–	Positive for *L. infantum*	
Indirect fluorescent antibody test IFAT for *L. infantum*	Positive 1:10,240	Positive 1:10,240	Positive 1:5120	
qPCR kDNA	Positive in whole blood (Ct 27) and Lymph node (Ct 17)	Positive in lymph node (Ct 28)	Negative in whole blood and positive in lymph node (Ct 26)	
Hematology				[36, 37]
RBC (10^6^/µL)	5.5	8.2	11	7.4–13.2
HGB (g/dL)	5.7	7.3	10.4	9.5–14.9
HCT (%)	25	35	41.7	22.5–42.7%
MCV (fL)	46	42	28	27.2–44.2
MCH (pg)	10.5	8.9	9.5	9.2–14.3
MCHC (g/dL)	22.5	21	25	28.6–36.4
WBC (× 10^3^/µL)	3.1	2.6	5.7	3.8–8.7
Band neutrophils (/µL)	0	0	0	0–2610
Segmented neutrophils (/μL)	1953	1508	3135	1200–5700
Lymphocytes (/μL)	868	702	1824	1000–2400
Monocytes (/μL)	217	364	285	210–790
Eosinophils (/μL)	31	26	399	60–990
Basophils (/μL)	31	0	57	10–190
Platelets (× 10^3^/µL)	348	789	440	206–605
Blood chemistry				[36, 37]
CPK (U/L)	562	213	239	37–623
AST (U/L)	89	56	67	25–75
ALT (U/L)	89	92	101	14–94
ALP (U/L)	20	16	22	17–93
GGT (U/L)	0.1	1.1	2.5	0–14
Total bilirubin (mg/dL)	0.5	0.5	0.6	0.2–1.5
Total proteins (g/dL)	6.8	7.6	6.9	6.2–7.9
Albumins (g/dL)	2	2.6	2.8	3.1–4.6
Total globulins (g/dL)	4.8	5	4.1	1.5–3.9
Albumin/globulin ratio	0.4	0.5	0.7	0–2.1
Cholesterol (mg/dL)	242	161	185	147–247
Triglycerides (mg/dL)	112	39	34	38–109
Amylase (U/L)	464	209	245	246–446
Lipase (U/L)	664	270	298	19–31
Urea (mg/dL)	74	43	57	21.8–61.3
Creatinine (mg/dL)	0.6	0.5	0.5	0.24–0.71
Na/K ratio	30	30	31	26.2–40.9
Serum protein electrophoresis				[35]
Albumin (%)	27.9	42.1	40.4	35.9–41.1
α-Globulins (%)	NA	10.9	11.6	–
α1-Globulins (%)	6	3.5	3.2	9–12
α2-Globulins (%)	10.7	7.5	8.4	8.4–14
β-Globulins (%)	28.9	27	27.6	–
β1-Globulins (%)	/	2.7	3.3	5.3–8.2
β2-Globulins (%)	–	8.6	8.8	17–21.8
β3-Globulins (%)	–	15.6	15.5	Our electrophoresis splits β2 and β3, while the reference groups them as β2
γ-Globulins (%)	26.5	20	20.4	5.2–10.4
Albumin (g/dL)	1.9	2.91	2.79	2.33–2.68
α1-Globulins (g/dL)	0.41	0.24	0.22	0.57–0.82
α2-Globulins (g/dL)	0.73	0.51	0.58	0.51–0.81
β1-Globulins (g/dL)	1.96	0.19	0.23	0.39–0.48
β2-Globulins (g/dL)	–	0.59	0.61	1.02–1.48
β3-Globulins (g/dL)	–	1.08	1.07	Our electrophoresis splits β2 and β3, while the reference groups them as β2
γ-Globulins (g/dl)	1.8	1.38	1.41	0.35–0.6
Total protein (g/dl)	6.8	6.9	6.9	5.8–7.4
Albumin/globulin ratio	0.39	0.73	0.68	0.68–0.77
C-reactive protein (mg/dL)	3.4	1.8	1	
Treatments	Treatment for leishmaniosis began on 9 September 2025 (day 5) with meglumine antimoniate (100 mg/kg, SC, SID) for 5 weeks, along with allopurinol (10 mg/kg/day PO) for 6 months	Treatment for ehrlichiosis was initiated on 21 October 2025 (day 48), with doxycycline (10 mg/kg/day PO) for 28 days	–	

*ALP* alkaline phosphatase, *ALT* alanine aminotransferase, *AST* aspartate aminotransferase, *CPK* creatine phosphokinase, *CRP* C-reactive protein, *GGT* gamma-glutamyl transferase, *HGB* hemoglobin, *HCT* hematocrit, *MCH* mean corpuscular hemoglobin, *MCHC* mean corpuscular hemoglobin concentration, *MCV* mean corpuscular volume, *RBC* red blood cells, *WBC* white blood cells

Soon after the diagnosis (day 5), a treatment protocol adapted from that commonly used in dogs [44] was initiated, consisting of meglumine antimoniate (100 mg/kg; Glucantime^®^, Boehringer Ingelheim) administered once daily, subcutaneously, for 5 weeks, in combination with allopurinol (10 mg/kg/day; Allopurinolo*®* Mylan) administered orally for 6 months. During therapy, the fox showed clear clinical improvement, with no treatment-related adverse effects, and urinalyses was performed periodically during follow-up to monitor xanthinuria.

 Subsequently, 4 weeks into the treatment schedule (day 33), the fox underwent a follow-up physical examination and laboratory testing. The skin lesions continued to improve, and the animal no longer showed generalized lymphadenomegaly or pale mucous membranes. Clinicopathological findings were also improved (Table 2). The fox tested negative on lymph node aspirate cytology, and the qPCR Ct value increased from 17 to 28, indicating a marked reduction in parasite load.

**Fig. 5:**
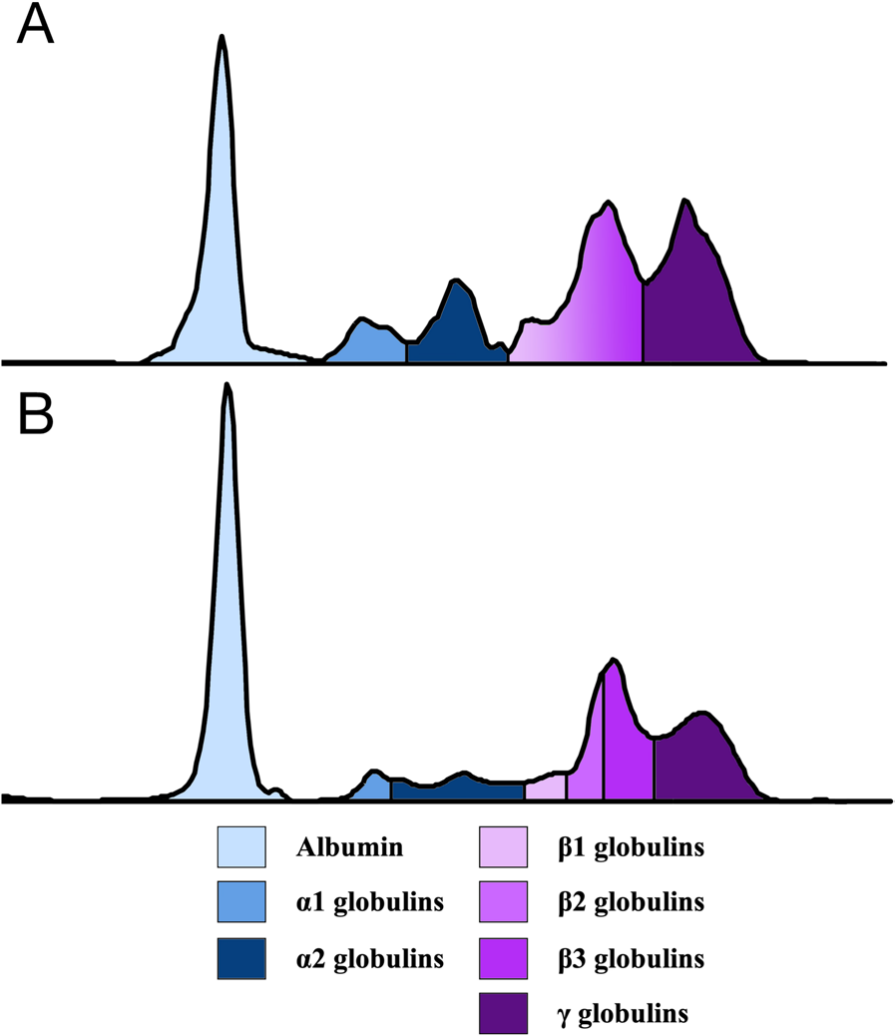
Serum protein electropherogram from a red fox (*Vulpes vulpes*) before (**A**; day 0) and after (**B**; day 68) treatment for leishmaniosis. The electrophoretic protein fractions, albumin, α1-, α2-, β1-, β2-, and γ-globulins, are shown from left to right and are represented by different colors in the figure legend

Given the clinical stability at this stage, therapy for ehrlichiosis was initiated on day 48 with doxycycline (10 mg/kg/day; Ronaxan^®^, Boehringer Ingelheim), administered orally for 4 weeks. The final assessment, conducted on day 68, revealed a continued overall improvement in the animal’s physical, hematobiochemical, serological, and molecular findings. Although a slight increase in serum globulins persisted and the qPCR Ct value decreased marginally from 28 to 26, the fox’s overall clinical condition markedly improved, and the animal gained weight (from 2.6 to 3.1 kg). The IFAT titer for *L. infantum* decreased to 1:5120, the SNAP Leish 4Dx^®^ test turned negative for *Ehrlichia* sp., CRP further declined from 1.8 to 1.0 mg/L, and serum protein electrophoresis showed pronounced normalization with a reduction in the γ-globulin peak and a less prominent β-globulin region, findings consistent with decreased immune activation and inflammation (Fig. 5).

### PBMC infection and cytokine gene expression analysis

**Fig. 6:**
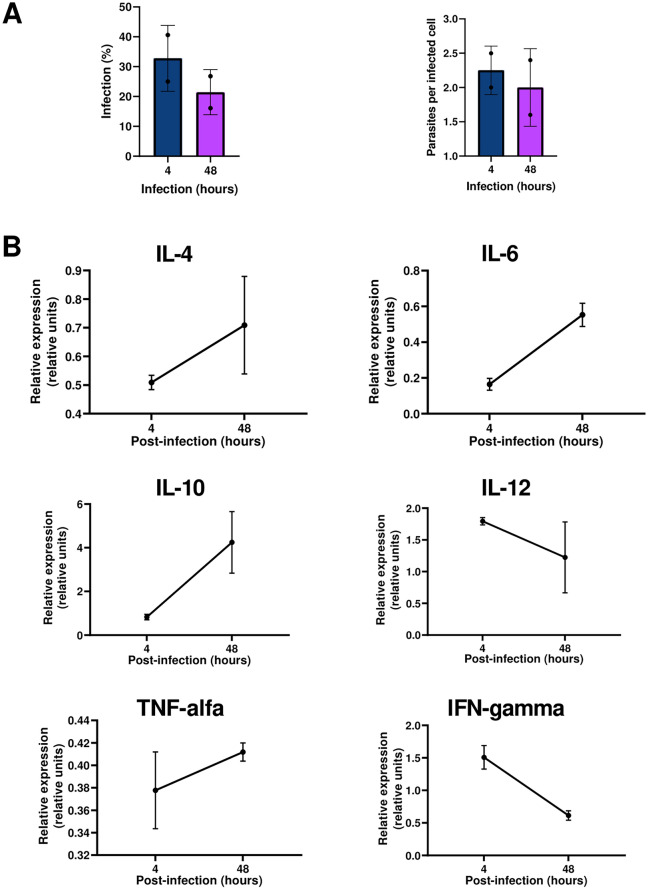
Percentage of infected red fox (*Vulpes vulpes*) macrophages and number of parasites per cell at 4 h and 48 h after infection with *L. infantum* (**A**). Relative gene expression of IL-4, IL-6, IL-10, IL-12, TNF-α, and IFN-γ quantified in monocyte-derived macrophages at 4 h and 48 h post-infection (**B**)

The percentage of infected macrophages decreased from 32.8% with an average of 2.25 parasites per infected cell at 4 h to 21.5% with an average of 2 parasites per infected cell at 48 h (Fig. 6A).

The relative gene expression of IL-4, IL-6, IL-10, IL-12, TNF-α, and IFN-γ quantified in monocyte-derived macrophages at 4 h and 48 h post-infection showed distinct temporal patterns (Fig. 6B). IL-4 expression showed similar levels between time points (i.e., 0.505 to 0.708 relative units at 4 h and 48 h, respectively). IL-6 and IL-10 expression levels increased over time, with IL-6 rising from 0.164 to 0.552 and IL-10 showing the highest overall expression, from 0.828 to 4.245. In contrast, IL-12 and IFN-γ expression levels decreased between time points (IL-12: 1.793 to 1.223; IFN-γ: 1.507 to 0.613). TNF-α expression remained low at both time points (0.377 and 0.411 relative units at 4 h and 48 h, respectively).

## Discussion

The data reported indicate that red foxes inhabiting endemic areas for *L. infantum* may become infected and develop clinical signs, probably owing to comorbidities or immunosuppressive events, as suggested by a cytokine gene expression pattern indicative of a nonprotective Th2 immune response (primarily through IL-10 upregulation). Moreover, we demonstrated a successful treatment protocol in a fox with improved clinical and laboratory findings after treatment (i.e., reduced antibody titers, decreased CRP levels, and negative lymph node cytology).

The molecular prevalence of *L. infantum* in red foxes (22% in live animals and 25% in dead animals) aligns with previous findings in southern Italy (20.8%, 10/48) [45]. This value also overlaps with the prevalence observed in sheltered dogs from hyperendemic areas of the same Apulia region (18%, 11/61) [46]. Eco-epidemiological conditions might explain the similar prevalence  observed between foxes and dogs, as sheltered dogs live in confined environments where sand flies are abundant and expose them to infection [47]. Similarly, foxes typically live in dens, which are suitable breeding sites for sand fly vectors, including *P. perniciosus* [48–50].

However, *L. infantum* prevalence data in red foxes show considerable variability across studies, with reported rates from 2.9% (7/244) [51], 40% (20/50) [52], up to 52% (48/92) [53] in Italy, as well as 45% (31/69) [54] in Spain and 59.5% (28/47) [55] in Greece. Nonetheless, data comparison is hindered by the source of the samples (i.e., necropsied animals) and by differences in the diagnostic methods employed [54, 56]. In addition, specific conditions, such as keeping hunting dogs in rural areas [57] and the synanthropic roaming behavior of foxes [58], may represent collateral risk factors for the parasite’s circulation.

The higher PCR sensitivity observed in spleen (25%; 5/20) compared with lymph node (15%; 3/20) tissue is consistent with previous studies conducted in dogs [59] and red foxes [54, 55], showing that the spleen is a preferred tissue for the detection of *Leishmania* in both healthy and sick animals. However, given the invasiveness of spleen biopsy and the risk of internal hemorrhage in animals following such a procedure [59], blood samples remain the most common tissue type collected from wild carnivorans, while conjunctival swabs could serve as a reliable, noninvasive alternative [60].

Considering the phylogenetic relationship between sequences herein obtained and those reported from dogs and sand flies, a dog–sand fly–fox biological circulation is suggested, given the high prevalence of *L. infantum* in canine populations in southern Italy [61], where *P. perniciosus* and *P. perfiliewi* are the primary vectors of *L. infantum* [62, 63]. In addition, sand flies commonly aggregate in structures housing domestic animals (e.g., sheep sheds, bird shelters, and dog kennels) and in rural settings [62], as well as in burrows and dens, including those made by red foxes [48]. All the environments above present a remarkably stable microclimate, enabling sand flies to live and breed year-round and thereby increasing opportunities for transmission. Although transmission from red foxes to sand flies has not yet been demonstrated, it remains plausible that infected foxes could serve as competent hosts, especially given evidence of infectiousness of other wild canid species [64, 65]. Such a role could help explain the limited effectiveness of control measures targeting only domestic dogs, such as culling infected individuals, as practiced in countries such as Brazil [66].

Apart from the present study, only one other investigation reported clinical alterations in necropsied red foxes in northern Spain, where 2 of 400 individuals showed skin lesions compatible with leishmaniosis [67]. In our dataset, the only seropositive fox using the SNAP Leish 4Dx^®^ was the sick one, whereas whole blood testing showed higher molecular positivity (21.9%; 9/41). Similar discrepancies between serological and molecular results have been reported in foxes from Spain [68] and Italy [53], with 2.1% (western blot) versus 14.1% positivity in the first case and 0% (IFAT) versus 52.2% in the second. Collectively, these data might suggest that red foxes are less susceptible to leishmaniosis, as has been frequently reported for other wildlife [2, 54, 56]. Nonetheless, some foxes may present overt disease, as demonstrated in the clinical case reported herein and in experimentally infected foxes [9]. In this case, the fox presented with desquamative and ulcerative dermatitis, along with clinicopathological alterations commonly reported in canine leishmaniosis and other carnivorans [21, 22, 69].

The clinicopathological findings, such as mild anemia, serum hyperglobulinemia with polyclonal β- and/or γ-globulinemia (mainly due to the increased antibody production), hypoalbuminemia (due to glomerular loss and/or inflammation), and increased CRP (due to an acute inflammatory status), suggest a chronic systemic infection and marked antigenic stimulation. On the basis of the clinicopathological findings, the fox was treated with meglumine antimoniate combined with allopurinol, which is recommended as first-line treatment in dogs [67] and has been used successfully in other wild carnivorans [22]. Another option, which could be considered when subcutaneous injection is not feasible, is oral administration of miltefosine in combination with allopurinol [44].

The absence of side effects and the effectiveness of the leishmanicidal treatment were reflected in improvements in general parameters (e.g., hematology and biochemistry) and diagnostic tests (e.g., IFAT titers, qPCR Ct values, and the disappearance of amastigotes on cytology), as well as in progressive improvement during follow-up. In this context, the use of meglumine antimoniate in combination with allopurinol avoided the need for repetitive cycles, which might be required in relapsed domestic dogs [70].

In this case, concurrent sarcoptic mange made it challenging to distinguish the overlapping clinical manifestations of the two conditions. For example, the generalized peripheral lymphadenomegaly is a frequent finding in canine leishmaniosis [44] but may also reflect a generalized reactive response to the diffuse skin inflammation common in sarcoptic mange [35]. The positive clinical outcomes were likely attributable to the anti-leishmanial treatment, which reduced the parasite burden and the associated inflammatory response, rather than the specific acaricidal treatment for sarcoptic mange. Moreover, treatment for ehrlichiosis was initiated after leishmaniosis because of persistently high serum gamma-globulin levels, which could reflect active ehrlichiosis, although molecular confirmation would be needed before starting doxycycline therapy.

The cytokine gene expression profile observed in *L. infantum*-infected red fox monocyte-derived macrophages indicates a modest immune activation during the first 48 h of infection. The low expression of IL-4 and IL-6 at both time points, despite the slight increase at 48 h, suggests that both pathways are moderately induced after parasite exposure. This pattern is consistent with previous findings in murine macrophages, where early IL-4 and IL-6 remain limited in the first 24–48 h of infection, with a more evident increase at later time points [71]. While red fox macrophages show a weak or delayed IL-6 response early after *Leishmania* exposure, dog macrophages tend to rapidly upregulate IL-6 (i.e., within the first 24 h) [39], suggesting differences in early inflammatory signaling that might be species-specific.

In contrast, the high expression of IL-10 aligns with the ability of *Leishmania* spp. to modulate host immunity shortly after cell contact, favoring the parasite’s survival inside the macrophage. A similar upregulation of this cytokine has been described in dogs, where its increased expression is correlated with higher parasite burden and progression to clinical disease [72–74].

Regarding the Th1 immune response, IL-12 peaked at 4 h, but declined substantially by 48 h, with persistently low TNF-α and decreasing IFN-γ levels. This pattern reflects a transient and potentially insufficient early Th1 response, which may limit macrophage activation. Comparable results have been reported in dogs during the initial stages of *L. infantum* infection, when both natural and experimental challenges often elicit weak or inconsistent IL-12 and IFN-γ induction in PBMCs [17, 75, 76].

Overall, the cytokine gene expression profile in *Leishmania*-infected red fox monocyte-derived macrophages suggests that red foxes may mount a limited immune response to the parasite. This could explain why red foxes often harbor *Leishmania* infection without developing overt clinical disease. In contrast, dogs are reported to mount a more dynamic and variable immune response, with the balance between pro-inflammatory and regulatory mechanisms ultimately determining whether they remain asymptomatic or develop clinical signs [74].

## Conclusions

Data from the present study strengthen the evidence that red foxes are involved in the sylvatic cycle of *L. infantum* in the Mediterranean basin, given their high infection rate and wide geographical distribution. Importantly, serological diagnostics and in vitro cytokine gene expression data may explain why most infected foxes remain subclinically infected. The presence of other comorbidities (e.g., sarcoptic mange) may complicate the interpretation of clinical presentation and delay treatment decisions in foxes, as in dogs. Moreover, this study presents the first case report of a sick fox, including treatment and follow-up, suggesting that the same standard therapeutic protocol used for dogs is effective in this animal species. Future studies should elucidate the infectiousness of sick and healthy *L. infantum*-infected red foxes to sand flies.

## Supplementary Information

Additional file 1: Sup. Info 1. Complete table with information for each red fox: ID, type of sampling, date of arrival, age, sex, geographical provenance, cause of admission, sample type, *Babesia* spp. and *Leishmania* spp. detections, and clinical presentations

## Data Availability

All data generated or analyzed during this study are included in this published article and its supplementary information files.

## Abbreviations

BLAST: Basic Local Alignment Search Tool
CBC: Complete blood cell count
cDNA: Complementary DNA
cPCR: Conventional polymerase chain reaction
CRP: C-reactive protein
Ct: Cycle threshold
DNA: Deoxyribonucleic acid
dqPCR: Duplex quantitative polymerase chain reaction
ELISA: Enzyme-linked immunosorbent assay
HCT: Hematocrit
HGB: Hemoglobin
*HSP70*: Heat shock protein 70
IFAT: Indirect fluorescent antibody test
IFN-γ: Interferon-gamma
IL-10: Interleukin-10
IL-12: Interleukin-12
IL-4: Interleukin-4
IL-6: Interleukin-6
ITS1: Internal transcribed spacer 1
kDNA: Kinetoplast DNA
MCH: Mean corpuscular hemoglobin
MCHC: Mean corpuscular hemoglobin concentration
MCV: Mean corpuscular volume
MOI: Multiplicity of infection
PBMC: Peripheral blood mononuclear cells
qPCR: Quantitative polymerase chain reaction
RBC: Red blood cells
RNA: Ribonucleic acid
TNF-α: Tumor necrosis factor-alpha
WBC: White blood cells
ZIMS: Zoological information management system
